# Bell County Medical Society, Minutes, Etc.

**Published:** 1886-04

**Authors:** R. P. Talley

**Affiliations:** Recording Secretary


### MINUTES

*Of the Second Monthly Meeting of the Bell County Medical Association Held in Dr. L. T. Battes Office, Belton, Texas, March* 3, 1886.

The Association was called to order by the President Dr. Frank Allen, of Belton.

On roll call the following gentlemen answered, to-wit. Drs. Frank Allen, H. C. Ghent, L. T. Batte, J. B. Fitzpatrick, Jos. W. Hunter, R. P. Talley and A. C. Enochs. Drs. R. R. Farr, A. S. Nunn, of Belton, and C. T. Simpson, of Temple, were present by invitation.

The work of the Association was very much interfered with by the unavoidable absence of Dr. Taylor Hudson, who was recording secretary pro tem, at the last monthly meeting, he having failed to send in the minutes as recorded by him.

Dr. S. Farrs petition for membership was duly received and referred to the Board of Censors.

Dr. Ghent read a paper by request, on "The Diagnostic Grunts of Children," for which the thanks of the Association were voted him by motion of Dr. Fitzpatrick.

Dr. Talley moved to amend motion adopted at the last monthly meeting to-wit. "That each member of this association is hereby earnestly requested to furnish the corresponding secretary with a complete list of his patrons who do not, will not, or can not, pay their bills for medical services in a reasonable time; these lists to be recorded by the corresponding secretary in a book for this purpose, and by him held subject to the inspection at any time, of any one contributing to the same," by adding that all practicing physicians in Bell county, be requested to contribute to this non-pay list of names, and that they, who do so contribute, be allowed the same privileges in regard to said lists, as if members in fact of the association. And that also the Belton Journal and the Temple Times (newspapers published weekly in Bell county) be paid from the funds of the association, to publish one time, that the Bell County Medical Association had, according to the first law of nature, to-wit: "self defense" adopted the policy as set forth in this motion and its amendment. This motion to amend prevailed, and Dr. Talley then read a long list of non-pay patrons. No other member present had a list ready to report, but all promised to report at next meeting. In the section on obstetrics, etc. Dr. Fitzpatrick reported a case of triplets, all boys, the second delivered still born, three distinct bags of water and one placenta. Dr. Hunter said that he had delivered a great many twins, and had never, in such cases, found only one placenta, or only one bag of waters; i. e. always a placenta and a bag of waters for each child.

The following gentlemen were appointed to report on sections at the next monthly meeting, to-wit: Practice of medicine, materia medica and pathology, Batte. Obstetrics and disease of women and children, Russell. Medical jurisprudence, Haley. State medicine, Fitzpatrick. Gynecology, Ghent. Association adjourned to meet 1st Tuesday in April next.

R. P. Talley,
Recording Secretary.

